# First evidence of long-term effects of transcranial pulse stimulation (TPS) on the human brain

**DOI:** 10.1186/s12967-021-03222-5

**Published:** 2022-01-15

**Authors:** Eva Matt, Lisa Kaindl, Saskia Tenk, Anicca Egger, Teodora Kolarova, Nejla Karahasanović, Ahmad Amini, Andreas Arslan, Kardelen Sariçiçek, Alexandra Weber, Roland Beisteiner

**Affiliations:** Imaging-Based Functional Brain Diagnostics and Therapy, Department of Neurology, High Field Magnetic Resonance Centre, Medical University of Vienna, Spitalgasse 23, 1090 Vienna, Austria

**Keywords:** Brain stimulation, Ultrasound, Transcranial pulse stimulation, Functional connectivity, Diffusion tensor imaging, Sensorimotor functions

## Abstract

**Background:**

With the high spatial resolution and the potential to reach deep brain structures, ultrasound-based brain stimulation techniques offer new opportunities to non-invasively treat neurological and psychiatric disorders. However, little is known about long-term effects of ultrasound-based brain stimulation. Applying a longitudinal design, we comprehensively investigated neuromodulation induced by ultrasound brain stimulation to provide first sham-controlled evidence of long-term effects on the human brain and behavior.

**Methods:**

Twelve healthy participants received three sham and three verum sessions with transcranial pulse stimulation (TPS) focused on the cortical somatosensory representation of the right hand. One week before and after the sham and verum TPS applications, comprehensive structural and functional resting state MRI investigations and behavioral tests targeting tactile spatial discrimination and sensorimotor dexterity were performed.

**Results:**

Compared to sham, global efficiency significantly increased within the cortical sensorimotor network after verum TPS, indicating an upregulation of the stimulated functional brain network. Axial diffusivity in left sensorimotor areas decreased after verum TPS, demonstrating an improved axonal status in the stimulated area.

**Conclusions:**

TPS increased the functional and structural coupling within the stimulated left primary somatosensory cortex and adjacent sensorimotor areas up to one week after the last stimulation. These findings suggest that TPS induces neuroplastic changes that go beyond the spatial and temporal stimulation settings encouraging further clinical applications.

## Background

Non-invasive brain stimulation (NIBS) has been shown to support brain functions in neurological and psychiatric disorders [1, 2]. Commonly used NIBS techniques, such as transcranial magnetic stimulation (TMS), or transcranial direct current stimulation (tDCS), for example, are based on electromagnetic effects on the brain that imply several limitations. First, due to electrical conductivity effects the spatial resolution is limited, meaning that, in addition to the actual stimulation site, also other brain areas are affected [3]. Second, brain stimulation with these techniques cannot access deep brain structures without affecting superficial layers [4].

In recent years, low intensity transcranial focused ultrasound (tFUS) has emerged as a NIBS method that overcomes these drawbacks. With a lateral resolution between 3 and 7 mm, tFUS allows precise brain stimulation of confined regions, for example, the primary somatosensory cortex [5–8]. tFUS stimulation of this area led to altered electrophysiological responses, such as attenuated somatosensory evoked potentials (SEPs) and modulated intrinsic oscillations in the beta frequencies [5, 6]. tFUS stimulation of subregions of the primary and secondary somatosensory cortex, individually located by using functional magnetic resonance imaging (fMRI), elicited transient tactile sensations in the hand contralateral to the stimulation site [7, 8] and produced evoked potentials at central and posterior electrodes (C3, P3; 8). Further, it has been demonstrated that tFUS facilitates brain functions specific to the brain stimulation target. For example, participants displayed better spatial and temporal tactile discrimination abilities after tFUS targeting the primary somatosensory cortex compared to sham stimulation [6]. tFUS of the primary motor cortex reduced reaction time in a stimulus response task indicating enhanced motor performance [9]. However, depending on the stimulation site, behavioral performance might be disrupted by tFUS as well. Legon et al. reported an impaired performance in a tactile spatial discrimination task due to the stimulation of the ventro-posterior lateral nucleus of the thalamus [10]. The corresponding P14 SEP component was inhibited demonstrating that tFUS can reach and modulate not only cortical tissue but also deep brain structures.

Recently, we introduced transcranial pulse stimulation (TPS) [11–13], a new NIBS technique that is based on single ultrashort ultrasound pulses (3 µs, repeated every 200–300 ms). With a lateral resolution comparable to tFUS, TPS allows a spatially distinct brain stimulation as well. TPS stimulates up to 8 cm into the brain reaching deep brain structures like the thalamus lying in a distance between 5 and 6.5 cm from the scalp [10]. The advantage of this method over tFUS is that tissue warming and standing waves [14–16] can be avoided due to the use of very short pulses without periodic waves or long sonication trains. Standing waves might lead to unintended secondary stimulation maxima limiting the spatial specificity of tFUS. Moreover, TPS is the first ultrasound-based NIBS technique that is approved for clinical applications (CE mark). By using magnetic resonance imaging (MRI) for neuronavigation, the application can be specifically adapted to individual brain anatomy and is monitored in real-time.

A first uncontrolled clinical feasibility study in patients with Alzheimer’s disease demonstrated that repeated TPS stimulation of specific cognitive networks improved memory and verbal functions up to three months [11]. These changes were mirrored by increased activation and functional connectivity within brain regions specific for mnestic functions as assessed with fMRI, as well as with increased cortical thickness [17]. In a sham-controlled electroencephalographic (EEG) study in healthy participants, we showed that focal stimulation of the cortical somatosensory representation altered SEP components specific to primary and secondary somatosensory processing. In addition, we observed a dose-dependent effect with the most effective stimulation setting applying 1000 pulses as compared to 10 or 100 pulses [11]. The application of TPS was well tolerated by the participants and patients. Side effects were rare and comprised transient headache or feelings of pressure at the stimulation site. No major side effects occurred and no signs of neuronal tissue damage, assessed with specific structural MRI sequences, were observed.

Besides effectiveness, safety and feasibility, the persistence of favorable effects is certainly critical for clinical applications. Up to now, little is known about the temporal dynamics and long-term effects of ultrasound-based NIBS. The majority of the tFUS studies evaluated neurophysiological online effects in humans [5–7, 9, 10, 18, 19] or short-term effects from minutes up to two hours in animal models [20, 21]. The first (uncontrolled) observation of persisting effects of ultrasound-based NIBS was provided by our clinical feasibility study that argues for functional activation and connectivity effects of TPS up to one week minimum and cognitive improvements up to three months minimum [11].

The major aim of this exploratory study was to probe first sham-controlled long-term effects of TPS and to investigate related neurophysiological mechanisms in healthy human participants. To this end, a randomized sham-controlled cross-over design was used with repeated sessions of verum and sham TPS targeting the cortical somatosensory representation of the right hand. Basic somatosensory processing was targeted, as this represents a well localizable function in the brain with well-established tests to assess perceptional functions (tactile spatial discrimination) and behavioral implications (sensorimotor abilities). Comprehensive MRI investigations were used to assess anatomical and functional alterations at the stimulation site and in connected sensorimotor networks. As we assume that TPS induces functional neuroplasticity by increasing the functional coupling between the stimulation site and connected brain areas, the primary outcome of this study represents the resting state functional connectivity in primary and secondary somatosensory networks. Ongoing functional up-regulation via NIBS might induce morphological changes, such as enlarged dendritic spines, increased synapse density, and modified interneural connections [22]. Thus, white matter structural integrity, assessed with diffusion-tensor imaging (DTI), and cortical volume, analyzed by using voxel-based morphometry (VBM), represent our secondary outcomes. Moreover, we expect that potential functional and structural alterations improve corresponding behavioral abilities such as tactile sensitivity and sensorimotor dexterity.

## Methods

### Study design

**Fig. 1:**
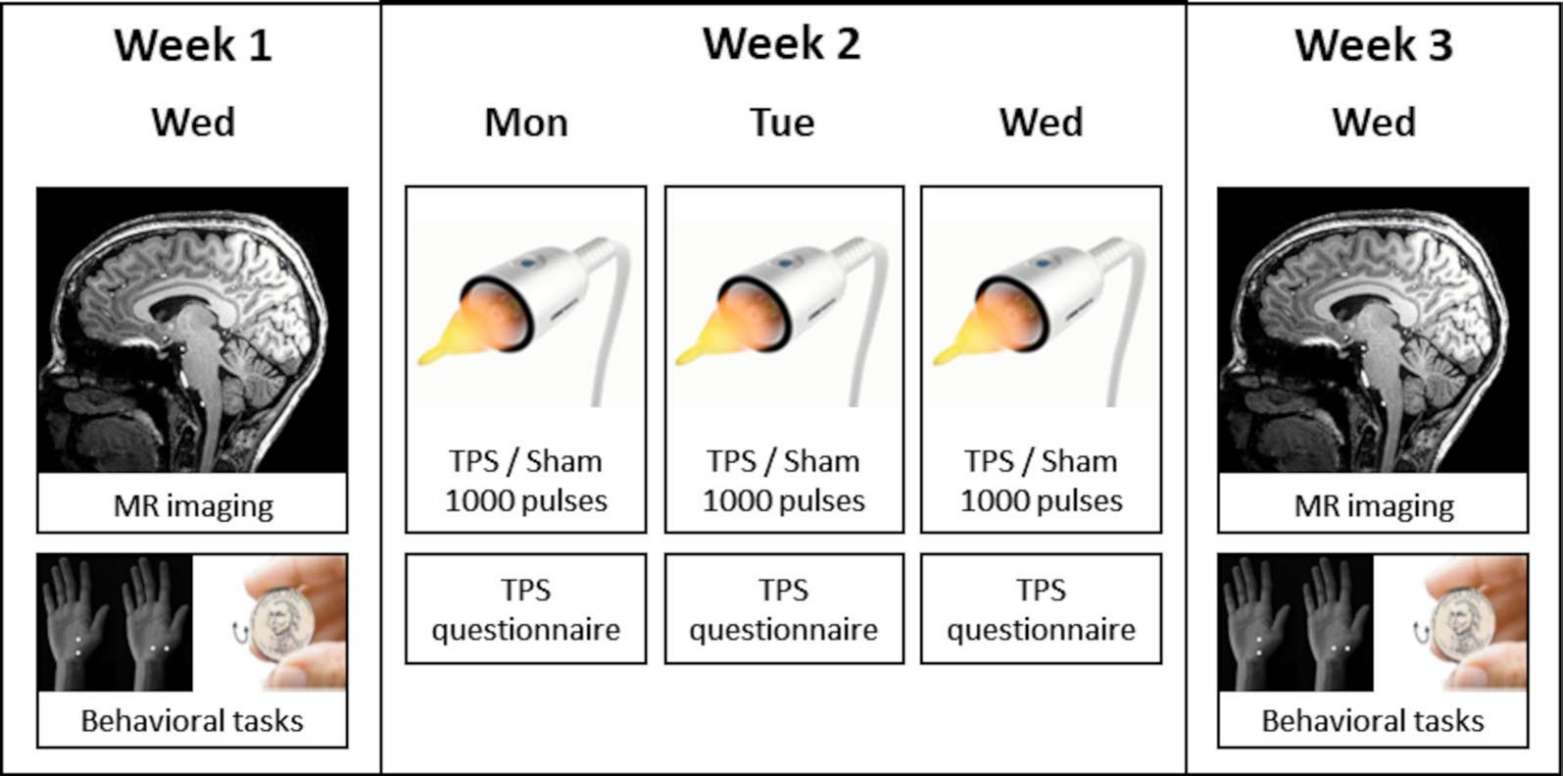
Longitudinal study design. An experimental block lasted three weeks with magnetic resonance (MR) imaging and behavioral tasks (2-point orientation discrimination [23] and coin rotation [24]) one week before and after transcranial pulse stimulation (TPS). Each subject received one block with sham and one block with verum TPS (three sessions on consecutive days) using a within-subject crossover design and one week pause between the blocks

This randomized, sham-controlled, and double-blind study was conducted at the Medical University of Vienna. Twelve healthy male participants with 18–35 years of age (mean age 26.50 years, SD = 5.00) that were right-handed and did not suffer from any neurological, psychiatric, or major somatic disease were recruited. For each participant, the study duration was seven weeks including one week pause between the two experimental blocks (3 weeks each, Fig. 1).

The baseline assessments in the 1 week of each block (week 1 and 5) comprised MR measurements and behavioral assessments of tactile and sensorimotor functions. In the second week of each block (week 2 and 6) three TPS interventions on three consecutive days were applied, either as real (verum) brain stimulation or as placebo (sham) stimulation. The MR and behavioral measurements were repeated in the third week of each block (week 3 and 7) to assess post-stimulation changes. Each subject received one block with sham and one block with verum TPS using a within-subject crossover design. The order of the experimental conditions was counterbalanced and randomly assigned. The experimenter applying TPS was informed about the actual condition (sham or verum), experimenters conducting MRI, safety evaluations, behavioral assessments, as well as data analysis were blinded.

### Transcranial pulse stimulation (TPS)

**Fig. 2:**
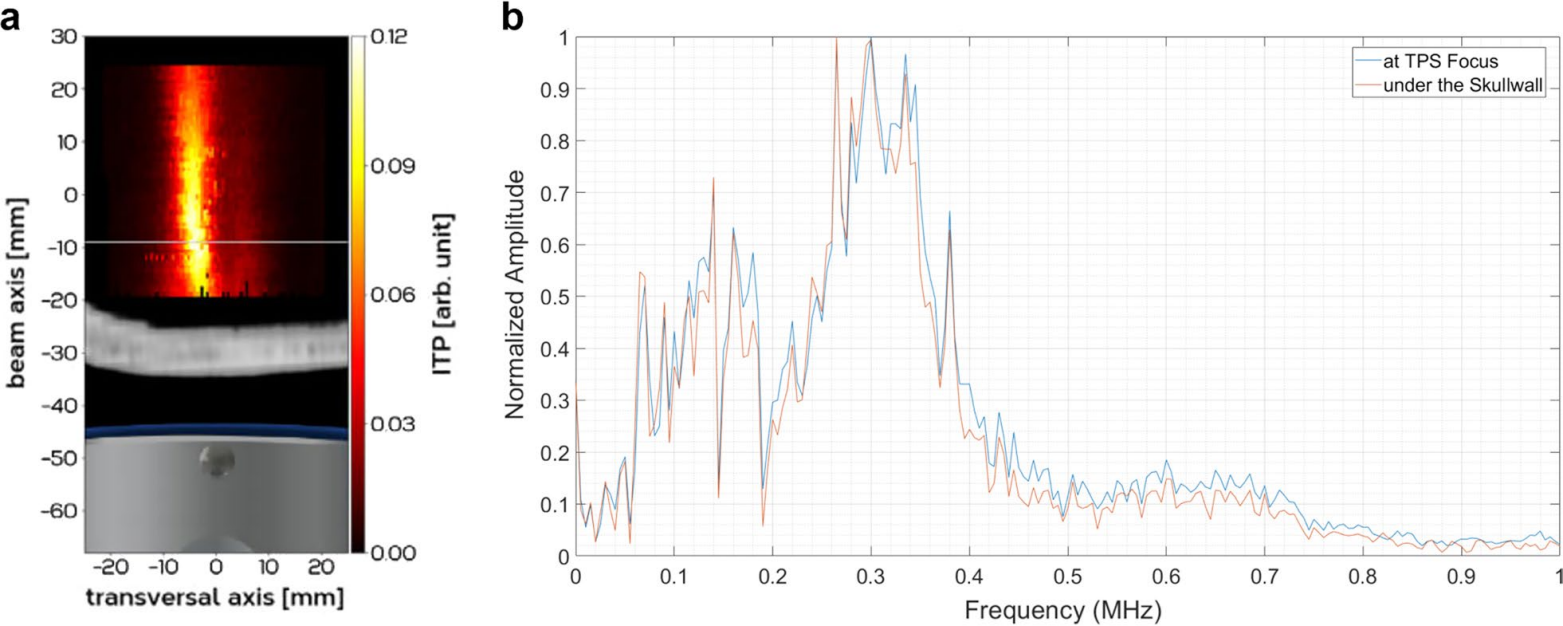
TPS pulse characterization. **a** Temporal-peak intensities (ITP) of a TPS pressure pulse through a human skull bone showing a highly focal transversal resolution of a few millimeters. **b** Fourier spectrum of a pressure pulse at TPS focus and under the skull demonstrating pressure attenuation through the skull across the frequency spectrum

TPS generates single ultrashort (3 µs) ultrasound pulses with typical energy flux densities of 0.2–0.3 mJ/mm^2^ and pulse repetition rates of 1–5 Hz (maximum spatial peak temporal average intensity I_SPTA_ = 100 mW/cm^2^, maximum spatial peak pulse average intensity I_SPPA_ = 111 W/cm^2^, maximum peak pressure = 25 MPa, mechanical index (MI) = 10.95). The I_SPTA_ fulfils the DIN EN 61689 norm, and the maximum peak pressure lies well below tissue damaging pressure levels (40 MPa, [25]). The US Food and Drug Administration (FDA) guidelines only exist for diagnostic, but not for therapeutic ultrasound [26]. tFUS studies typically exceed diagnostic limits in one or more parameters [27]. While the I_SPPA_ for TPS lies within the FDA limits for cephalic use (I_SPPA_ = 190 W/cm^2^), the I_SPTA_ is marginally higher (I_SPTA_ = 94 mW/cm^2^) and the MI exceeds respective FDA limits (MI = 1.90) [26]. However, comprehensive animal studies exist for therapeutic ultrasound applications and have been used for the successful clinical certification process of TPS [13]. Indeed, TPS is clinically certified as therapy for Alzheimer’s disease (CE mark). Comprehensive simulations and measurements of the temporal-peak intensities for free water, human skull and brain sample are provided by our preceding work [11]. Figure 2a shows measurements of the temporal-peak intensities field of a pressure pulse trough a human skull bone demonstrating a high transversal resolution of the acoustic focus. The human skull produces a temporal-peak intensity drop of 80–90% [11] across the frequency spectrum (Fig. 2b).

**Fig. 3:**
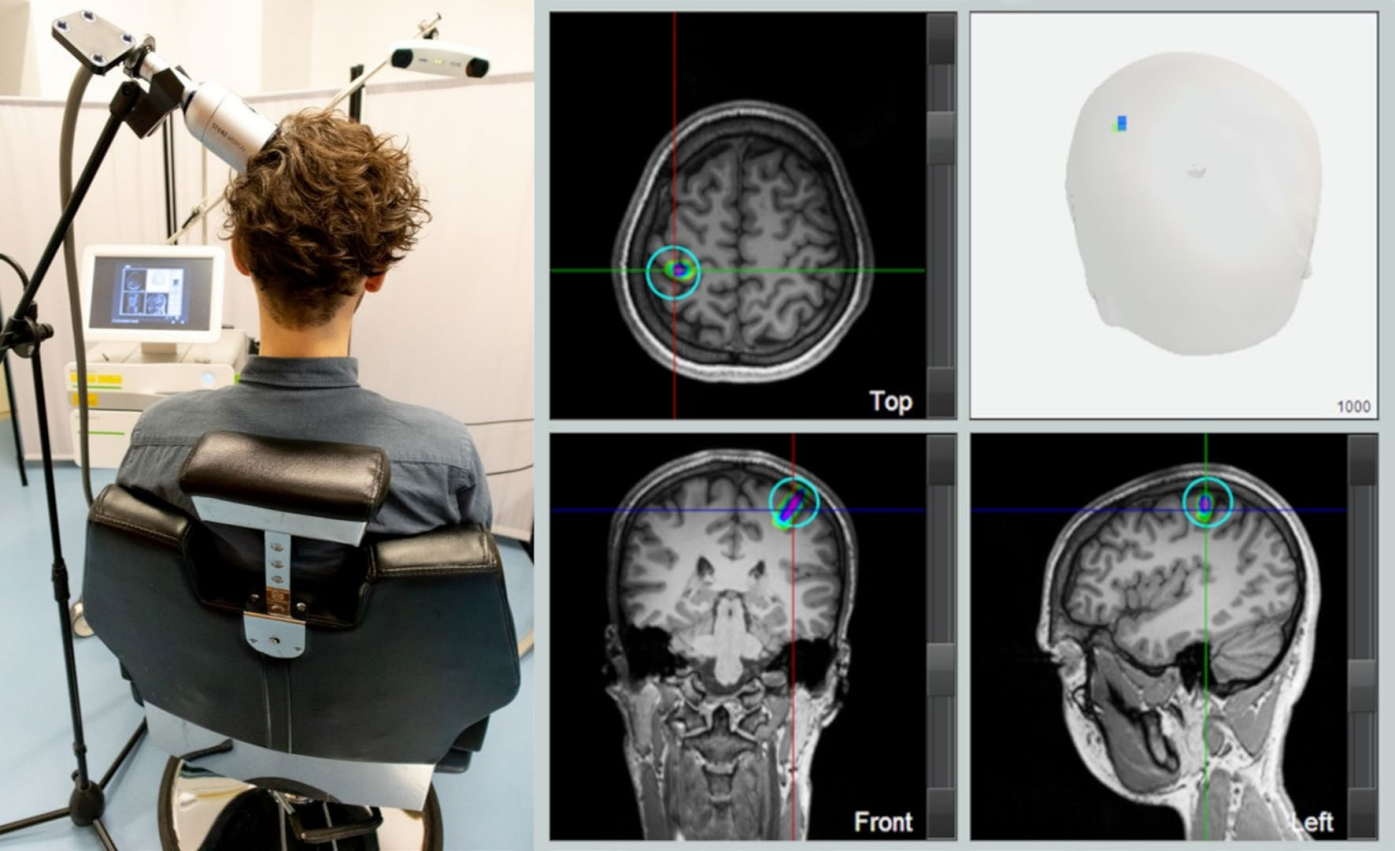
Focal transcranial pulse stimulation (TPS). TPS setup included a pulse generator device, a touch screen for real-time neuronavigation and an infrared camera system tracking the positions of the handpiece and the head of the participant via goggles affixed with infrared markers (left). The TPS handpiece was fixed using a tripod with a clamp focusing the ultrasound beam on the cortical primary somatosensory representation of the right hand, in the left postcentral gyrus posterior to the individual sigmoidal hook sign (marked by a turquoise circle). The TPS pulses of one session in a representative subject are displayed on the reconstructed head surface and in top, front and left orientation of the individual brain anatomy, showing the lowest (green) to highest pulse density (magenta)

For the current investigation, the TPS handpiece was fixed with the ultrasound beam focused on the cortical primary somatosensory representation of the right hand, in the left postcentral gyrus posterior to the individual sigmoidal hook sign (Fig. 3). The participants were seated in a comfortable armchair with the head laid on a restricting headrest. A tripod with a clamp was used to fix the handpiece to the participant’s head. Exact positioning was achieved by MR-based real-time neuronavigation including an infrared camera system that tracked the positions of the handpiece and the head of the participant via goggles affixed with infrared markers (Fig. 3). Plenty of bubble-free ultrasound gel (Aquasonic Clear, Parker Laboratories) had to be applied to cover the skin and hair at the stimulation area to avoid acoustic impedance borders. Using TPS parameters as defined by a pilot experiment [11], 1000 TPS pulses (energy flux density = 0.25 mJ/mm^2^, pulse repetition rate = 4 Hz) were applied in each TPS session that lasted approximately 4 min. Sham stimulation was achieved by blocking the ultrasound beam with a sham cap on the TPS handpiece that looked identical and produced a similar knocking sound as the verum stimulation. After each TPS session, participants were asked about sensations, potential side effects and a subjective estimation if sham or verum TPS was applied.

### Structural MRI

MR measurements were conducted at a 3 Tesla Siemens Prisma MR scanner using a 64-channel head coil and comprised anatomical, functional resting state, diffusion-tensor imaging (DTI), as well as clinical standard scans. Brain structural anatomy was assessed using a T1-weighted MPRAGE sequence with a spatial resolution of 1 mm isotropic (TE/TR = 2.7/1800 ms, inversion time = 900 ms, flip angle = 9°). These anatomical scans were used for TPS neuronavigation and for volumetric analyses (voxel-based morphometry, VBM). A T2-weighted fluid-attenuated inversion recovery (FLAIR) sequence (TE/TR = 100/10000 ms, inversion time = 2500 ms, flip angle = 160°) and a T2-weighted 2D-fast-low-angle shot (FLASH) sequence (TE/TR = 19.9/690 ms, flip angle = 20°) were applied to detect potential lesions, edemas or bleedings.

VBM analyses were performed using the SPM toolbox Computational Anatomy Toolbox CAT12 (http://www.neuro.uni-jena.de/cat/). VBM preprocessing included segmentation for longitudinal data, an estimation of the total intracranial volume, and smoothing (8 mm FWHM kernel) using CAT12 default values. On second level, segmented data were compared between the post stimulation sessions (referenced to the respective pre stimulation scan) with the intracranial volume as a covariate to account for different brain sizes. VBM analysis was applied to whole-brain data as well as for the grey matter volume within regions of interest (ROIs) of the Neuromophometrics atlas implemented in CAT12.

### Resting state functional connectivity

For the resting state scan, a whole-brain T_2_*-weighted gradient-echo-planar imaging (EPI) sequence was applied (TE/TR = 35/1400 ms, flip angle = 90°, in-plane acceleration = GRAPPA 2, multiband acceleration factor = 2, resolution = 2 mm isotropic). During the resting state measurement, the participants were required to think of nothing in particular while fixating a visually presented cross. The resting state scan lasted approximately 10 min (430 volumes). Resting state data analyses were performed with the CONN toolbox v19c [28] and included default preprocessing comprising realignment, unwarping, slice-time correction, structural segmentation, normalization, outlier detection (ART-based scrubbing) and smoothing (8 mm FWHM kernel). Denoising was achieved using a band-pass filter [0.008–0.09 Hz], removal of motion confounds (6 motion parameters and their first derivatives), removal of white matter and cerebrospinal fluid signals (five principal components extracted from the cerebrospinal fluid and the white matter masks) and scrubbing. For first level analysis, a bivariate correlation of the corrected time series of all voxels was calculated. On second level, the graph theoretical measure global efficiency (GE) was analyzed for the left- and right-lateralized sensorimotor network. GE is defined as the inverse of the shortest path length between each pair of nodes of the network and represents the capacity for parallel information processing within a network [29]. The sensorimotor network comprised the primary motor and somatosensory cortex (precentral and postcentral gyri), the secondary somatosensory cortex (left parietal operculum [30]), and higher-order somatosensory integration areas (superior parietal lobe, supramarginal and angular gyri, superior lateral occipital cortex). These anatomical ROIs were defined according to the Harvard–Oxford-atlas as implemented in the CONN toolbox. On group level, global efficiency was compared between the post stimulation sessions referenced to the respective pre stimulation scans (correlation coefficient 0.35, false discovery rate (FDR) 0.05 corr.).

### Diffusion tensor imaging (DTI)

DTI data were acquired using a whole-brain 64-direction EPI sequence (TE/TR = 95/10500 ms, multiband acceleration factor = 2, resolution = 2 mm isotropic, b-value = 1000 s/mm^2^). DTI indices fractional anisotropy (FA), mean diffusivity (MD), as well as axial (AD) and radial diffusivity (RD) were investigated to comprehensively assess alterations of the white matter microstructure on a whole-brain level and within sensorimotor ROIs. FA is a measure for the coherence of water diffusion direction with higher values indicating better white matter integrity [31, 32]⁠. MD is the mean rate of free water diffusion independent of the directionality [32]. AD measures the rate of water diffusion along the principal axis of diffusion, i.e., the underlying fiber orientation, and reflects axon number and caliper [33]⁠, while RD is the magnitude of water diffusion perpendicular to the white matter tract indicating myelin changes [34]. Intact neuronal microstructures, such as axonal cell membranes and myelin sheaths, displace intra- and extracellular water leading to lower MD, AD and RD values [31, 32]⁠.

**Fig. 4:**
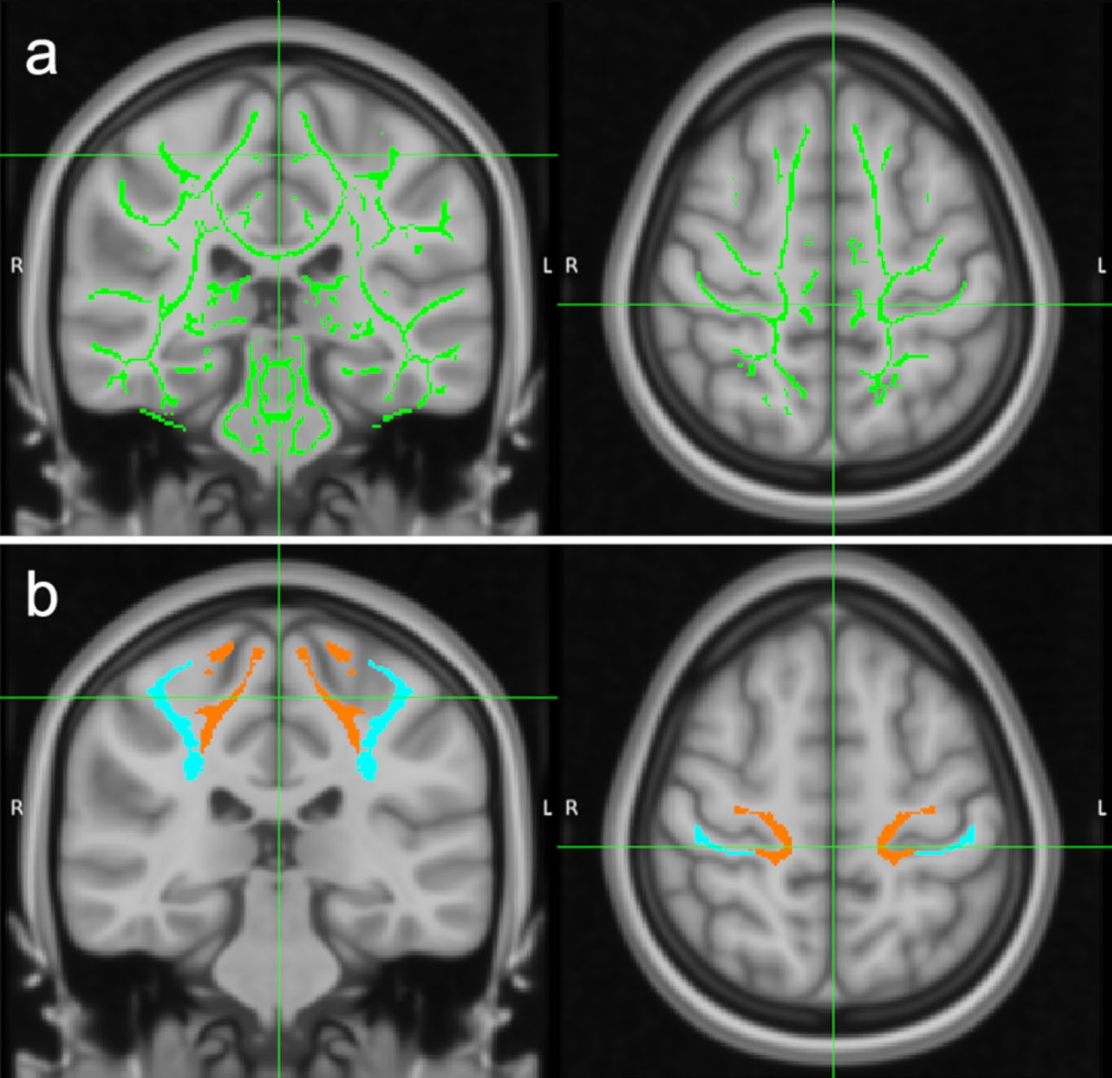
Diffusion tensor imaging (DTI) analysis. **a** For whole-brain white matter data analysis the Tract-based Spatial Statistics (TBSS) white matter skeleton (green) was used. **b** For the regions of interest analysis, left primary somatosensory (S1, blue) and the left primary motor (M1, orange) white matter regions, derived from the Human Sensorimotor Tracts Labels atlas, were used

DTI data preprocessing and statistical analyses were performed using FSL 5.0.9 and related toolboxes. Data preprocessing included extraction of brain tissue from the b0 volume using the FSL Brain Extraction Tool (BET, threshold 0.1), eddy current correction using the FMRIB’s Diffusion Toolbox 3.0 and smoothing of DTI images using fslmaths with a 1-voxel box kernel an the f-median flag as recommended for longitudinal data [35]. Subsequently, DTI indices were reconstructed using DTI-FIT. The resulting images were further processed with Tract-based Spatial Statistics (TBSS) [36]. After the removal of outliers, the FA images of all four sessions of a participant were coregistered to a subject-specific template using the TBSS registration with the -n flag option. Subsequently, the coregistered images were normalized to MNI standard space using FMRIB58_FA template. The resulting mean FA map was thinned to create an average white matter tract skeleton using the default threshold 0.2 and individual FA values were projected onto the mean skeleton (Fig. 4a). By using the registration and skeletonization warps as well as the skeleton projection vectors derived from the TBSS processing of the FA images, the MD, AD and RD images were similarly processed.

Statistical evaluation of all DTI indices projected onto the white matter skeleton was done using FSL randomise with the threshold-free cluster enhancement option (5000 permutations, family-wise error (FWE) 0.05 corr.). As for within-subject comparisons using FSL randomise a one-sample t-test against 0 is recommended, the contrast of interest [(verum post vs. verum pre) vs. (sham post vs. sham pre)] was computed with fslmaths first. For the ROI-analyses of DTI indices, the Human Sensorimotor Tracts Labels [37] implemented in FSLeyes was used to create the left primary somatosensory (S1) and left primary motor (M1) white matter ROIs (Fig. 4b). The accurate location of these ROIs was visually inspected by overlaying the ROIs on the individual white matter segments derived from the SPM segmentation procedure. The mean values for FA, MD, AD, and RD were extracted within these ROIs and statistically analyzed using SPSS v26. As ROI data were not normally distributed, a non-parametric Wilcoxon test was applied to compare pre- and post-stimulation values for both conditions (FDR 0.05 corr.).

### Behavioral assessments

After the MR measurements, behavioral assessments of tactile and sensorimotor functions were performed. The tactile spatial discrimination threshold was measured using a 2-point orientation discrimination task [23]. Here, a caliper with a given tip separation distance was applied at the participant’s thenar eminence of the right hand in either horizontal or vertical orientation. The participant was not able to see the caliper and should indicate if the horizontally oriented stimulus preceded or followed the vertical stimulus. Nine distances between 0 and 10 mm (each repeated eight times) were tested in randomized order. The proportion of correct responses was assessed and the spatial threshold for 75% correct responses served as the main outcome variable. After the 2-point orientation discrimination task, the participants underwent a coin rotation task as a measure for manual dexterity and sensorimotor processing [24]. The coin rotation task was shown to be related to functional activation in the primary somatosensory cortex [38] and predicts fine hand movements relevant for activities of daily living [39]. The subjects were asked to flip a 2€ coin along the horizontal axis with their right hand as fast as possible and time needed for 20 coin turns (180 degree flips) was recorded. Behavioral tasks were tested and validated in ten pilot subjects beforehand. Behavioral data were analyzed with SPSS v26 using a factorial design with the within-subject factors condition (sham/verum) and session (pre/post stimulation).

### Correlation analyses

The relations between neurophysiological measures demonstrating a TPS effect and behavioral scores (2-point-orientation discrimination, coin rotation) were examined by correlation analyses. As data were not normally distributed, a non-parametric Spearman’s rank correlation analysis was applied. Previous literature demonstrated an non-linear relation between age and DTI indices [40]. To account for this potentially confounding between-subject difference, age was controlled for by applying a partial Spearman’s correlation analysis.

## Results

### Functional connectivity

**Fig. 5:**
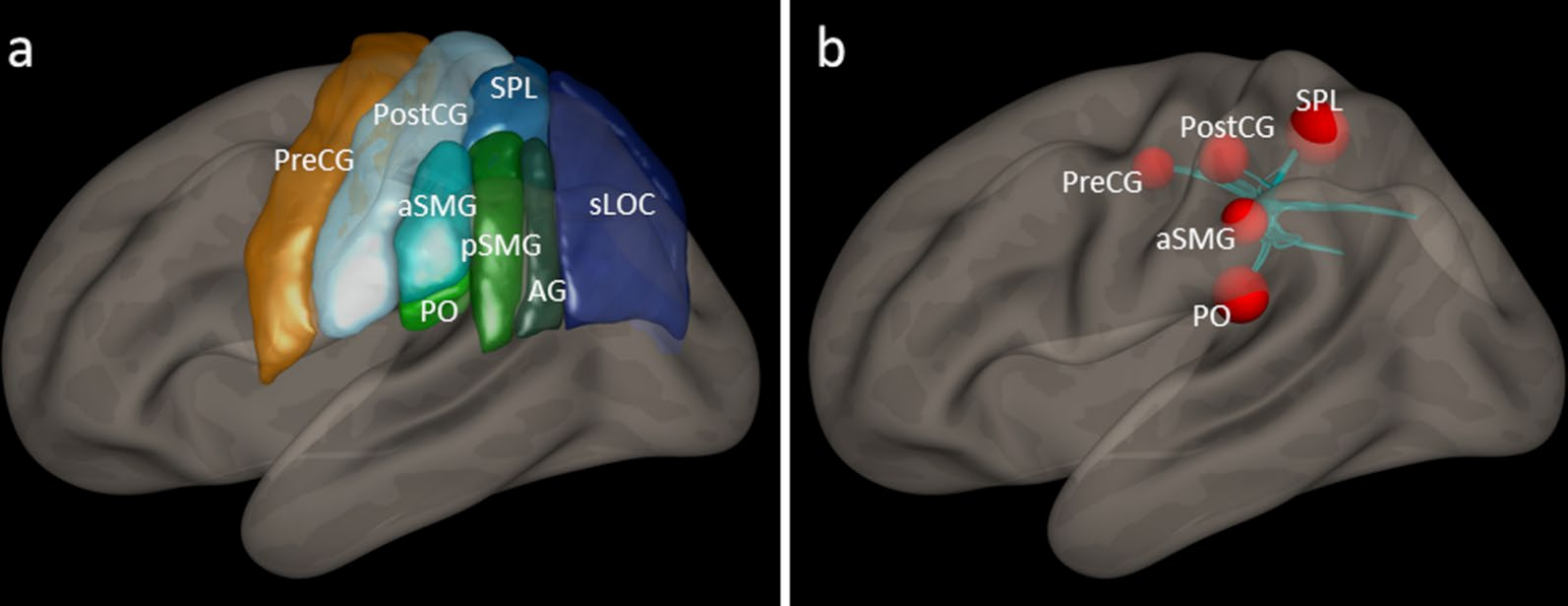
Resting state global efficiency of the sensorimotor network. **a** The sensorimotor network comprised the precentral gyrus (PreCG), postcentral gyrus (PostCG), superior parietal lobule (SPL), anterior supramarginal gyrus (aSMG), posterior supramarginal gyrus (pSMG), angular gyrus (AG), superior lateral occipital cortex (sLOC), and the parietal operculum (PO). These anatomical ROIs were defined according to the Harvard–Oxford-atlas. **b** In the stimulated left-hemispheric sensorimotor network, global efficiency values were significantly higher in the verum compared to the sham condition. Significant hubs within the network are represented by red spheres weighted according to the T value for the contrast between the conditions (FDR 0.05 corr.). No effects were detected in the non-stimulated right hemisphere

Global efficiency (GE), as the capacity for parallel information processing within a network [29], was significantly higher in the verum condition in the stimulated left (p = 0.040), but not in the non-stimulated right (p = 0.210), sensorimotor network. Significantly different hubs within the left-hemispheric sensorimotor network comprised precentral and postcentral gyri, the superior parietal lobule, the anterior supramarginal gyrus and the parietal operculum (Fig. 5, Table 1).

**Table 1:** Global efficiency values in the somatosensory network and its regions of interest

	Sham		Verum		Verum vs. Sham* T-value (p-value, FDR-corr.)
	Pre mean (SD)	Post mean (SD)	Pre mean (SD)	Post mean (SD)	
Network	0.573 (0.161)	0.458 (0.198)	0.477 (0.175)	0.512 (0.184)	1.93 (0.040)
PreCG l	0.588 (0.256)	0.496 (0.241)	0.497 (0.262)	0.560 (0.193)	2.13 (0.045)
PostCG l	0.613 (0.135)	0.482 (0.233)	0.483 (0.199)	0.584 (0.189)	2.54 (0.037)
SPL l	0.661 (0.194)	0.498 (0.255)	0.512 (0.230)	0.611 (0.188)	3.16 (0.033)
aSMG l	0.658 (0.211)	0.529 (0.277)	0.527 (0.219)	0.597 (0.151)	2.32 (0.040)
pSMG l	0.582 (0.195)	0.467 (0.192)	0.548 (0.155)	0.434 (0.258)	n.s
AG l	0.439 (0.210)	0.350 (0.208)	0.453 (0.101)	0.346 (0.279)	n.s
sLOC l	0.465 (0.249)	0.387 (0.199)	0.396 (0.196)	0.453 (0.211)	n.s
PO l	0.575 (0.155)	0.458 (0.289)	0.399 (0.297)	0.510 (0.234)	2.83 (0.036)

PreCG l: left precentral gyrus, PostCG l: left postcentral gyrus, SPL l: left superior parietal lobule, aSMGl: left anterior supramarginal gyrus, pSMG l: left posterior supramarginal gyrus, AG l: left angular gyrus, sLOC l: left superior lateral occipital cortex, PO l: left parietal operculum, n.s.: not significant

^*^For comparing the conditions, the contrast (verum post vs. pre stimulation) vs. (sham post vs. pre stimulation) was computed

### White matter microstructure

**Fig. 6:**
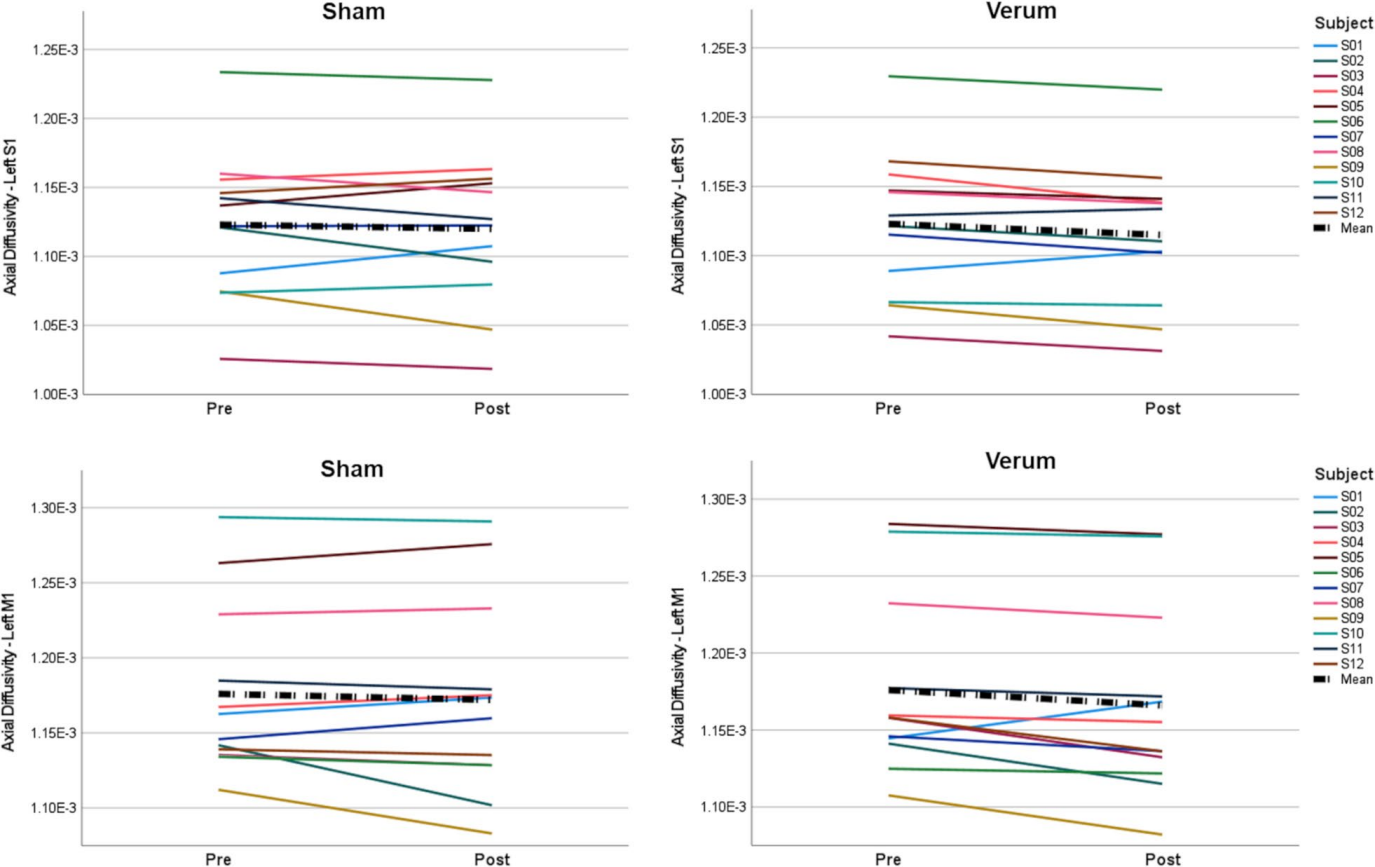
Diffusion tensor imaging indices. Axial diffusivity (AD) values in the left primary somatosensory (S1, upper row) and left primary motor (M1, lower row) white matter regions of interest one week before (Pre) and one week after (Post) the sham and verum TPS applications. Data is depicted for individual subjects (S01-S12) and as mean over all subjects (black line). AD values significantly decreased after the verum stimulation, indicating an improved axonal status in the stimulated sensorimotor network

TBSS analyses of DTI indices (FA, MD, AD, RD) did not show significant effects in the white matter tract skeleton. In contrast, ROI analysis revealed significantly reduced AD within the white matter tracts in the primary somatosensory ROI (p = 0.034, FDR-corr.) and primary motor ROI (p = 0.038, FDR-corr.) after verum TPS compared to the respective baseline (non-parametric Wilcoxon-tests, two-tailed, n = 12, Table 2). On individual level, AD values decreased in the majority (11/12 for the primary motor ROI, 10/12 for the primary somatosensory ROI) of the subjects after the verum stimulation, while after no coherent change was observable after sham (Fig. 6). No significant effects were found for the other DTI indices (FA, MD, RD), or for the sham condition (Table 2).

**Table 2:** DTI indices in the left primary sensory and primary motor white matter

	Left S1				Left M1			
	Sham		Verum		Sham		Verum	
	Pre	Post	Pre	Post	Pre	Post	Pre	Post
FA	3.895E−01	3.883E−01	3.885E−01	3.870E−01	3.878E−01	3.865E−01	3.864E−01	3.864E−01
	(3.587E−02)	(3.707E−02)	(3.786E−02)	(3.672E−02)	(3.144E−02)	(3.403E−02)	(3.290E−02)	(3.202E−02)
MD	7.913E−04	7.898E−04	7.920E−04	7.874E−04	8.509E−04	8.480E−04	8.523E−04	8.435E−04
	(5.269E−05)	(5.039E−05)	(5.048E−05)	(5.058E−05)	(5.786E−05)	(5.760E−05)	(5.972E−05)	(5.720E−05)
AD	1.123E−03	1.120E−03	1.123E−03^a^	1.115E−03^a^	1.176E−03	1.172E−03	1.176E−03^a^	1.166E−03^a^
	(5.328E−05)	(5.614E−05)	(5.243E−05)	(5.165E−05)	(5.681E−05)	(6.521E−05)	(5.781E−05)	(6.202E−05)
RD	7.212E−04	7.199E−04	7.227E−04	7.195E−04	7.846E−04	7.811E−04	7.866E−04	7.779E−04
	(5.186E−05)	(4.754E−05)	(4.950E−05)	(4.906E−05)	(6.197E−05)	(5.962E−05)	(6.327E−05)	(5.927E−05)

Data as mean (SD) for the DTI indices fractional anisotropy (FA), mean diffusivity (MD), axial diffusivity (AD) and radial diffusivity (RD)

S1—primary somatosensory white matter region of interest, M1—primary motor white matter region of interest

^a^Significant comparison between pre and post stimulation data (Wilcoxon test, FDR 0.05 corr.)

### Volumetric and morphological analyses

Volumetric analysis of the grey matter using voxel-based morphometry (VBM) did not show significant effects of TPS, neither on a whole-brain level nor on ROI-level. Safety evaluations of the clinical structural sequences (T2-weighted FLAIR and FLASH images) did not reveal any signs of bleedings, edema, or other morphological alterations after the sham or verum interventions.

### Sensorimotor functions

For the coin rotation task as a measure for manual dexterity and sensorimotor processing, factorial analysis did not show significant effects for the factors (n = 12, condition: p = 1.000; session: p = 0.416) and their interaction (p = 0.195). On a descriptive level, time needed for 20 coin rotations remained at about the same level after the sham intervention (pre stimulation: 11.75 ± 1.39 s, post stimulation: 11.83 ± 1.37 s), but decreased 0.75 s on average in the verum stimulation (pre stimulation: 12.17 ± 2.20 s, post stimulation: 11.42 ± 1.58 s).

Performance in the 2POD task as an indicator for tactile acuity improved slightly in the post-stimulation test (verum: 3.53 ± 3.24 mm; sham: 3.16 ± 2.58 mm) compared to the respective baseline (verum: 4.73 ± 3.03 mm; sham: 4.66 ± 3.01 mm) for both, verum and sham condition. However, these observations did not reach statistical significance (n = 12, condition: p = 0.665, session: p = 0.130, condition*session: p = 0.876).

### Correlations between behavioral and neurophysiological variables

Correlation analysis between significant neurophysiological variables (resting state GE, AD in the primary somatosensory and motor ROI) and behavioral tests did not reveal significant results. However, a trend between AD in the motor ROI and 2POD performance points to a subtle relation between improved axonal status and tactile acuity (rank correlation coefficient ρ = 0.281, p = 0.056, n = 48, see Table 3).

**Table 3:** Spearman’s rank correlation analysis between neurophysiological and behavioral variables

	AD –S1 l	AD –M1 l	GE – Network	GE – PreCG l	GE – PostCG l	GE – SPL l	GE – aSMG l	GE –PO l
CR								
ρ	−0.132	− 0.214	− 0.051	− 0.042	− 0.001	− 0.173	− 0.031	0.077
p	0.377	0.149	0.733	0.775	0.993	0.240	0.834	0.604
2-POD								
ρ	− 0.159	0.281	− 0.040	− 0.094	− 0.100	− 0.116	− 0.051	− 0.105
p	0.286	0.056	0.787	0.525	0.500	0.434	0.733	0.479

CR, coin rotation; 2-POD, 2-point orientation discrimination; AD, axial diffusivity; GE, global efficiency; PreCG l, left precentral gyrus; PostCG l, left postcentral gyrus; SPL l, left superior parietal lobule; aSMG l, left anterior supramarginal gyrus; pSMG l, left posterior supramarginal gyrus; AG l, left angular gyrus; sLOC l, left superior lateral occipital cortex; PO l, left parietal operculum; ρ, rank correlation coefficient

### Sensations during TPS stimulation

On average, the participants’ estimation if sham or verum TPS was applied was correct to 69% (n = 72), whereby the accuracy improved slightly in the second experimental block (72%, n = 36) compared to the first block (67%, n = 36). The subjective certainty of the condition assignment, rated between 1 (not certain) and 10 (very certain), increased steadily from 5.58 ± 2.64 (n = 12) in the first TPS session to 7.33 ± 1.87 (n = 12) in the 6th session. Sensations at the scalp were frequently reported, with tactile impressions (e.g., “knocking”) being common in both conditions (Table 4). Feelings of pressure and pain at the scalp were more frequently reported in the verum condition; however, the mean intensity was rated quite low (between 2 and 3 out of 10). Besides one single report about twitching of the left thighs during a TPS verum session, no peripheral sensations were noted by the participants.

**Table 4:** Ratings of sensations at the scalp during TPS stimulation

	Sham		Verum	
	n (%)	Mean intensity (SD)	n (%)	Mean intensity (SD)
Tactile	7 (9.72)	3.64 (2.01)	7 (9.72)	4.67 (3.20)
Pressure	3 (4.17)	3.00 (1.73)	11 (15.28)	2.23 (1.54)
Pain	1 (1.39)	2.00 (0.00)	12 (16.67)	2.27 (1.47)

Number (n) and percentage of sessions in which specific sensations occurred out of all 72 TPS sessions (12, participants, 6 session each). Mean and standard deviation (SD) for sensation intensity, ranging from 1 to 10 (= max. intensity), were calculated for reported sensations only

## Discussion

Using repeated TPS applications in healthy participants, we provide the first sham-controlled evidence of long-term effects of ultrasound-based NIBS on human brain structure and function. One week after the last TPS stimulation of the cortical somatosensory hand representation, global efficiency in the sensorimotor network of the stimulated left hemisphere significantly increased. Further, TPS improved white matter microstructure in the stimulated left sensorimotor regions.

For feasible and effective clinical applications of NIBS, persisting changes of brain functions and associated symptom relief are required. Contrary to investigations of online-effects of tFUS [5–7, 9, 10, 18, 19], this work addresses long-term neuroplastic changes induced by ultrasound-based NIBS persisting at least one week. Indeed, we were able to demonstrate increased global efficiency in the stimulated sensorimotor network one week after verum TPS compared to sham stimulation. This functional upregulation was not only observable in the stimulated left primary somatosensory cortex and directly interconnected regions (secondary somatosensory cortex, motor cortex), but extended to higher integrative areas (anterior supramarginal gyrus, superior parietal lobule). These findings indicate that repeated applications of verum TPS lead to a long-term increase of the functional coupling between somatosensory processing areas, integrative and motor regions.

Previous NIBS studies have demonstrated that increased functional connectivity within a network induced by brain stimulation is related to improved behavioral functions or clinical symptoms supported by this network. For example, upregulation of the motor network connectivity by intermittent theta-burst stimulation was correlated with symptom relief in patients with upper limb paresis [41] and increased functional connectivity of the precuneus by high-frequency rTMS was related to improved episodic memory in patients with Alzheimer’s disease [42]. Similarly, we demonstrated that increased functional connectivity within the memory network after TPS was correlated with improved cognitive functions in patients with Alzheimer’s disease [11]. These behavioral improvements persisted up to three months, indicating a long-lasting effect of TPS on brain functions. However, as this first clinical TPS feasibility study was not sham-controlled, placebo and training effects might have contributed to the improvements as well. With the current controlled investigation, we demonstrated a functional and structural upregulation in networks specific to the stimulation site for verum TPS in comparison to sham stimulation. Thus, the hypothesis that TPS induces functional and structural plasticity is supported by the current findings.

In contrast to our initial hypothesis, functional up-regulation was not found to be significantly correlated with behavioral improvements in this study. This lack of significant enhancement of tactile acuity and manual dexterity after TPS might be caused by statistically underpowered behavioral tests due to the small sample size, learning and ceiling effects regarding sensorimotor functions in healthy participants. Yet, functions corresponding to the stimulation site might be influenced by early, direct effects of ultrasound NIBS [6]. Possibly, sensorimotor tests during or immediately after the stimulation might reveal a short-term benefit of TPS.

After the verum TPS stimulation, we found decreased AD in the left primary motor and left primary somatosensory ROIs, while it remained unchanged after sham stimulation. Although TPS stimulation was confined to the somatosensory cortex, both ROIs displayed a comparable AD decline after stimulation. Mirroring functional connectivity results, that demonstrate a global efficiency increase in a widespread sensorimotor network, microstructural integrity seems to be promoted in primary motor areas and potentially in other regions that are highly interconnected with the stimulated somatosensory cortex. In the developing brain, axial and radial diffusivity decrease as indicators for axonal and myelin formation, respectively [33]⁠, while in advanced age and due to neurodegenerative processes, diffusivity indices (MD, AD, RD) typically increase while FA is reduced [43]. In the current investigation, repeated focal application of TPS decreased AD in healthy human white matter that might reflect an increased brain fiber density or enlarged axonal calipers [33, 44]. Potentially, the regenerative benefit of TPS is even higher for pathological tissue, supporting its application in neurodegenerative disorders like Alzheimer’s disease, Parkinson’s disease and Multiple Sclerosis. There was a non-significant trend for a positive correlation between improvements in axonal status and tactile acuity that needs to be confirmed by follow-up studies.

Like in the clinical feasibility study in patients with Alzheimer’s disease [11], repeated TPS stimulation was well tolerated by the participants. While the TPS handpiece was continuously moved along the skull in the clinical feasibility study, TPS was applied focally and stationary in the current investigation. Still, sensations reported at the stimulation site were transient and the intensity of pressure and pain ratings was low. Contrary to previous reports about tactile impressions in peripheral regions induced by tFUS of the corresponding cortical representation [7], no stimulation-specific peripheral sensations were reported by the participants in the current study. Further, no signs of swellings, bleedings or lesions were observed after the stimulation, as comprehensively assessed with structural MR imaging including volumetric analyses. These data suggest that repeated application of TPS, even in a highly focal manner, is safe and well tolerated by the participants.

Ratings directly after the TPS interventions imply that the participants were able to distinguish between sham and verum condition to a certain extent, particularly after repeated applications. This is in contrast with our observations with moving TPS handpiece applications, where AD patients’ differentiation capability was close to chance. Presumably, sensory impressions like knocking, pressure and slight pain at the stimulation site informed the participants about the actual condition. However, a direct effect of the subjective condition assignment on the outcome variables, particularly on resting state functional connectivity and white matter microstructure, seems unlikely. Still, stimulation settings minimizing sensory impressions at the stimulation site should be tested for future studies.

As the current investigation focused on long-term TPS effects, immediate effects of the stimulations were not assessed. However, online or short-term effects during or directly after the stimulation could be informative about requirements and extent of further neuroplastic reorganization. Thus, future studies should include longitudinal assessments starting immediately after the stimulation to estimate the temporal dynamics of TPS effects on brain and behavior.

## Conclusions

Repeated TPS targeting the primary somatosensory cortex increased functional coupling and improved white matter structural integrity in the sensorimotor network up to one week after the stimulation. These findings suggest that TPS induces neuroplastic changes that go beyond the spatial and temporal stimulation settings. This evidence of sham-controlled long-term effectivity, safety and feasibility encourages further clinical applications of TPS.

## Data Availability

The datasets used and/or analyzed during the current study are available from the corresponding author on reasonable request.

## Abbreviations

2POD: 2-Point orientation discrimination
AD: Axial diffusivity
DBS: Deep brain stimulation
DTI: Diffusion-tensor imaging
EEG: Electroencephalography
FA: Fractional anisotropy
FDR: False discovery rate
FDA: Food and Drug Administration
FLAIR: Fluid-attenuated inversion recovery
FLASH: Fast-low-angle shot
fMRI: Functional magnetic resonance imaging
FWE: Familywise error
GE: Global efficiency
I_SPTA_: Spatial peak temporal average intensity
I_SPPA_: Spatial peak pulse average intensity
MD: Mean diffusivity
MI: Mechanical index
MRI: Magnetic resonance imaging
NIBS: Non-invasive brain stimulation
RD: Radial diffusivity
ROI: Region of interest
SEPs: Somatosensory evoked potentials
tDCS: Transcranial direct current stimulation
tFUS: Transcranial focused ultrasound
TMS: Transcranial magnetic stimulation
TPS: Transcranial pulse stimulation
VBM: Voxel-based morphometry
